# Tumour growth rate and invasive interval cancer characteristics in a UK breast cancer screening population

**DOI:** 10.1007/s00330-024-11342-x

**Published:** 2025-01-21

**Authors:** Muzna Nanaa, Roido Manavaki, Thiemo J. A. van Nijnatten, Natalia Stranz, Serena Carriero, William A. Coleman, Iris Allajbeu, Nicholas R. Payne, Elisabetta Giannotti, Sarah E. Hickman, Otso Arponen, Fiona J. Gilbert

**Affiliations:** 1 Department of Radiology, School of Clinical Medicine, University of Cambridge, Cambridge, UK; 2 Department of Radiology, Royal Bolton Hospital, Bolton, UK; 3 Department of Radiology and Nuclear Medicine, Maastricht University Medical Center, Maastricht, The Netherlands; 4 GROW School for Oncology and Reproduction, Maastricht University Medical Center, Maastricht, The Netherlands; 5 Cambridge Breast Unit, Cambridge University Hospitals NHS Foundation Trust, Cambridge, UK; 6 Department of Radiology, Barts Health NHS Trust, The Royal London Hospital, London, UK

**Keywords:** Breast, Interval cancer, Mammography

## Abstract

**Objectives:**

To estimate tumour volume doubling time (TVDT) of interval cancers (ICs).

**Methods:**

Two radiologists retrospectively reviewed prior screening and diagnostic mammograms and measured mean diameter on “visible” ICs. Univariate analyses of clinicopathological variables (ER, HER2, grade, age at diagnosis, and breast density) were undertaken, and those with *p* < 0.1 were included in a generalised linear model to estimate TVDT, cancer size at screening, and time of cancer visibility for “non-visible” tumours.

**Results:**

From 2011 to 2018, 476 ICs were diagnosed, almost half in the third year after screening with 86% grade 2 or 3. A visible abnormality at screening was identified in 281/476 (59%) cases. Significant differences in TVDT were found with age (*p* < 0.02), ER status (*p* < 0.0001). Median TVDTs of grade 1, 2 and 3 cancers were 317, 288, and 195 days, respectively (*p* < 0.001). For non-visible cancers, the median estimated size at screening was 1.7 mm (IQR 1.0–2.5) for grade 1, 2.5 mm (IQR 1.5–5.9) for grade 2, and 0.9 mm (IQR 0.4–2.0) for grade 3 cancers, *p* < 0.001. The estimated time for cancer visibility was 489 days (IQR 229–682) after screening and 645 days (IQR 527–798) for cancers diagnosed in the third year after screening.

**Conclusion:**

Using TVDT of retrospectively visible interval cancers, non-visible interval cancer sizes can be estimated at the time of screening. Increasing the frequency of screening from three-yearly to two-yearly invitations would reduce the number of interval cancers significantly.

**Key Points:**

***Question*** *Growth modelling of visible interval cancers (ICs) at screening helps to track the likely progression of non-visible ICs over the screening interval.*

***Findings*** *Tumour doubling time of visible ICs at screening is positively associated with age and ER status and inversely associated with cancer grade.*

***Clinical relevance*** *Interval cancer characterisation and growth modelling can be helpful to better predict the benefits of supplemental screening and the frequency of screening, given a minimum detectable size.*

## Introduction

Breast cancer screening programmes are designed to detect breast cancer early and in a cost-efficient manner. The frequency of imaging is determined from observational studies with economic modelling used to balance the costs against the effectiveness. In the UK, the triennial screening programme detects 67% of breast cancers in females aged 50–70 years who attend screening [1]. The number of interval cancers (ICs) is an important quality measure of a screening programme in addition to the traditional quality measures (tumour type, size, and grade) of screen-detected cancers [2]. ICs represent a heterogeneous group of cancers detected after a negative screening mammogram and before the next scheduled examination. These cancers are regularly reviewed for educational purposes and to determine if there has been a “failure” of screening. Most ICs are too small to be detected by mammography at the time of the screening examination, particularly with longer three-yearly screening intervals.

On a retrospective review of screening mammograms, ICs can be classified as “visible” or “non-visible”. Visible ICs can show either suspicious malignant features (false negatives), subtle malignant features (minimal signs of malignancy), or benign-looking features (true negatives). The non-visible cancers (mammogram normal) at screening are either fast-growing incident cancers that occur during the screening interval (true interval cancers), or they could be mammographically occult or masked by breast density [3, 4]. Breast density is a risk factor for developing breast cancer, and dense breasts increase the risk of IC due to the masking effect [5, 6].

ICs have less favourable histopathological characteristics and prognosis than screen-detected cancers [7, 8]. Hovda et al observed no differences in histopathological characteristics between ICs classified as true interval cancers, cancers with minimal signs of malignancy, and missed cancers [9]. According to Weber et al, missed ICs showed a more favourable histologic grade than true ICs and were less frequently triple-negative cancers [10]. MacInnes et al reported a shorter tumour volume doubling time (TVDT) for oestrogen receptor (ER)-negative, grade 3, age below 60 years in ICs classified as very subtle or suspicious [11].

Tumour growth analysis is important to determine what steps might be needed to reduce ICs and improve breast cancer screening programmes. The growth rate of different types of cancers found in the population of screening age will help inform the economic models of frequency of imaging and be of practical value in recommending changes to reduce interval cancers and reduce the rate of next-round screen-detected cancers that potentially could have been found by a more sophisticated test at the previous round. In the UK, the National Health Service Breast Screening Programme (NHSBSP) offers women aged 50–70 years a triennial full-field digital mammography. This study aims to report the characteristics of interval cancers, analyse the TVDT of the visible cancers at screening according to patient age, breast density, IC category, cancer grade, and hormonal receptor status in an interval cancer cohort. Clinicopathological factors found to affect TVDT in this real-world observational data from a single screening programme were subsequently applied to estimate TVDT of non-visible cancers, their size at screening and the time point at which they might have become visible.

## Materials and methods

### Study patients

**Fig. 1:**
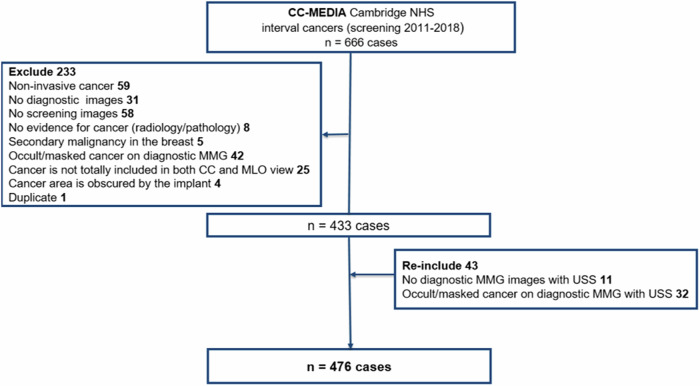
A flow chart shows the number of initial interval cancer cases and the cases that were ruled out. CC, craniocaudal; MLO, mediolateral oblique; MMG, mammogram; NHS, National Health Service, USS, ultrasound

This retrospective study used anonymised mammograms from one NHSBSP centre under ethical approval (Health Research Authority Research Ethics Committee (HRA REC) 20/LO/0104, HRA Confidentially Advisory Group (CAG) 20/CAG/0009, and Public Health England (PHE) Research Advisory Committee (RAC) BSPRAC_090). The Cambridge Cohort–Mammography East-Anglia Digital Imaging Archive (CC-MEDIA) dataset includes cases from the Cambridge and Huntingdon, and Norwich Breast Screening programmes. A subset of CC-MEDIA, consecutively collected ICs between January 2011 and December 2018 from different mammography vendors, with more than 90% from Philips Healthcare and the remainder from Hologic, GE Healthcare, and Sectra AB, was used. ICs, defined as cancers diagnosed in between two screening rounds: within 40 months of the previous screening examination and not detected at the subsequent screening round, were included. The ground truth was histopathology and information including tumour grade, ER and human epidermal growth factor-2 (HER2) status was obtained from histopathology reports. Non-invasive cancers, cases where screening mammograms were not available or when the cancer region was not totally included on both craniocaudal (CC) and mediolateral oblique (MLO) views were excluded. In cases with no available diagnostic mammogram or when measurements could not be taken because of occult disease or masking on the diagnostic mammogram, ultrasound size collected from ultrasound reports was used, where this was available, Fig. 1.

### Image analysis

Two dedicated breast radiologists (one reader with 8 years of breast imaging experience (M.N.) and one of five readers (N.S., S.C., E.G., I.A., and T.N.) with between 3 and 13 years of experience in breast imaging) retrospectively reviewed the prior screening mammograms and diagnostic mammograms of ICs in consensus to determine if the cancer was visible in hindsight. The second reader was chosen based on availability, and an unbiased approach through random rotation. In case of disagreement, arbitration with a third reader took place. ICs were classified as either visible (benign, minimal signs of malignancy, or suspicious) or non-visible at screening mammography (mammogram normal). Cancers were classified into three categories based on their features on screening mammography: ‘Benign-looking cancers’ exhibited only benign features (true negatives), ‘cancers with minimal signs of malignancy’ showed subtle malignant features, which were not sufficient to warrant a recall, and ‘suspicious cancers’ displayed obvious malignant features that were missed by readers (false negatives). Breast Imaging Reporting and Data System (BI-RADS) 5th edition density score (A, B, C, and D) of the screening mammograms and of the area where the cancer later developed (non-dense = BI-RADS A and B or dense = BIRADS C and D) were also reported. Tumour size was measured on screening, if visible, and diagnostic mammograms [(longest diameter + perpendicular diameter)/2] using the same view for both time points, either craniocaudal (CC) or mediolateral oblique (MLO), whichever was larger.

### TVDT calculation (visible cancers at screening)

**Fig. 2:**
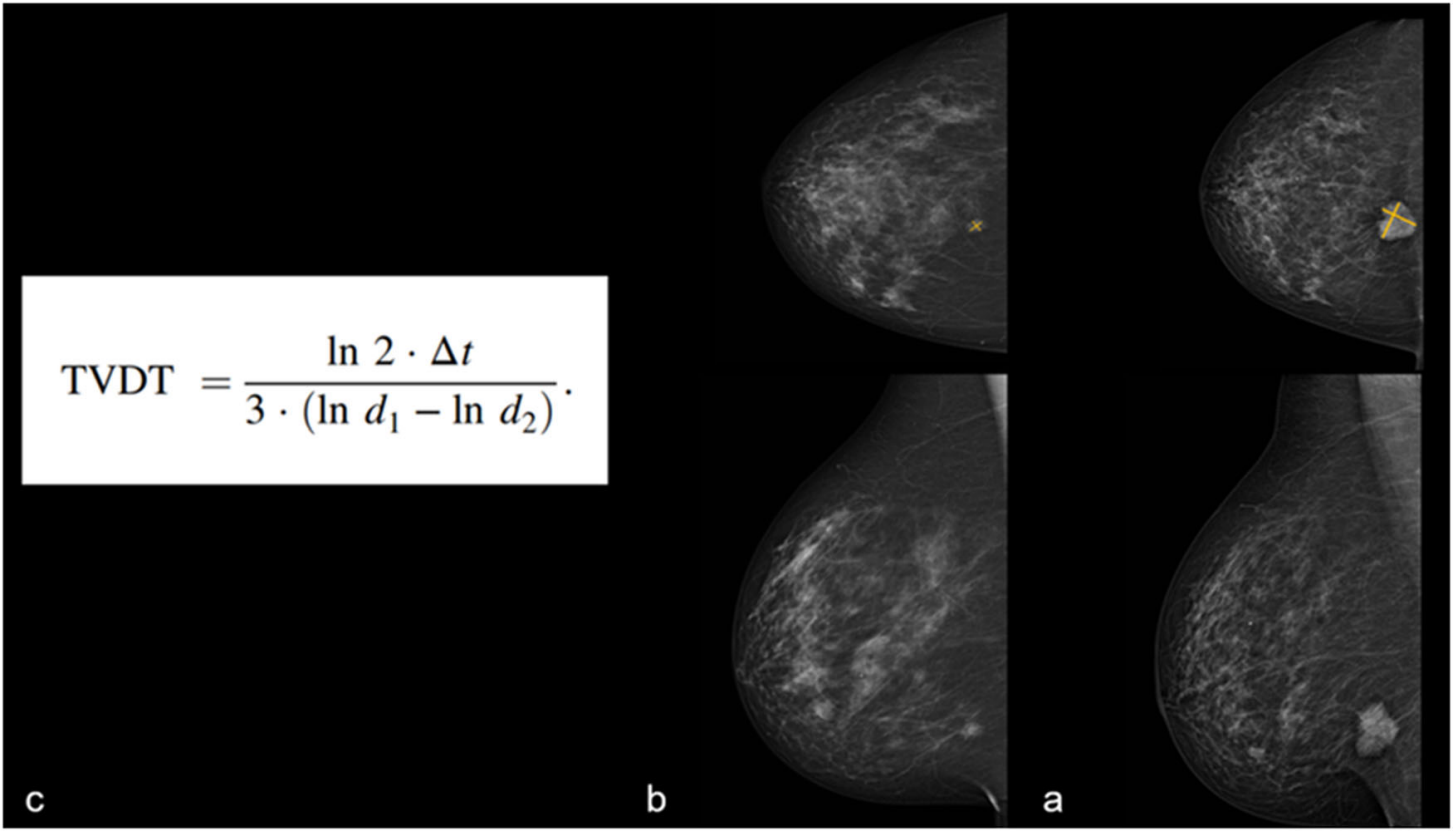
Visible interval cancer at the time of screening: A 60-year-old female was diagnosed in the second year (931 days) following the screening with a 31 mm grade 3 ER-positive HER2 negative IDC with HGDCIS and LCIS. Using the same view (MLO view) on both diagnostic (**a**) and screening (**b**) MMG, mean size was measured based on the longest diameter and the true perpendicular diameter. Based on the mean size at both time points and the time interval in days (**c**), the calculated TVDT for this case was 230 days. HGDCIS, high-grade ductal carcinoma in situ; IDC, invasive ductal carcinoma; LCIS, lobular carcinoma in situ; MMG, mammogram; TVDT, tumour volume doubling time

TVDT was calculated for visible cancers at screening using the equation [ln (2) × Δt / 3 × [ln (d1) − ln (d2)]], where Δt refers to the time between screening and diagnostic mammography (days), d1is the mean diameter at diagnostic mammography (mm), d2 is the mean diameter at screening mammography (mm) [12, 13]. For cases with no diagnostic mammogram or those where measurements could not be taken because of occult or masked cancer, diagnostic mammography size was imputed by fitting a linear regression of ultrasound size on the corresponding mammography size at diagnosis for the visible cancers. The mammography size was subsequently estimated from the ultrasound size at diagnosis for non-visible cancers. Figure 2 illustrates the TVDT calculation of visible IC at screening.

### TVDT estimation (non-visible cancers at screening)

**Fig. 3:**
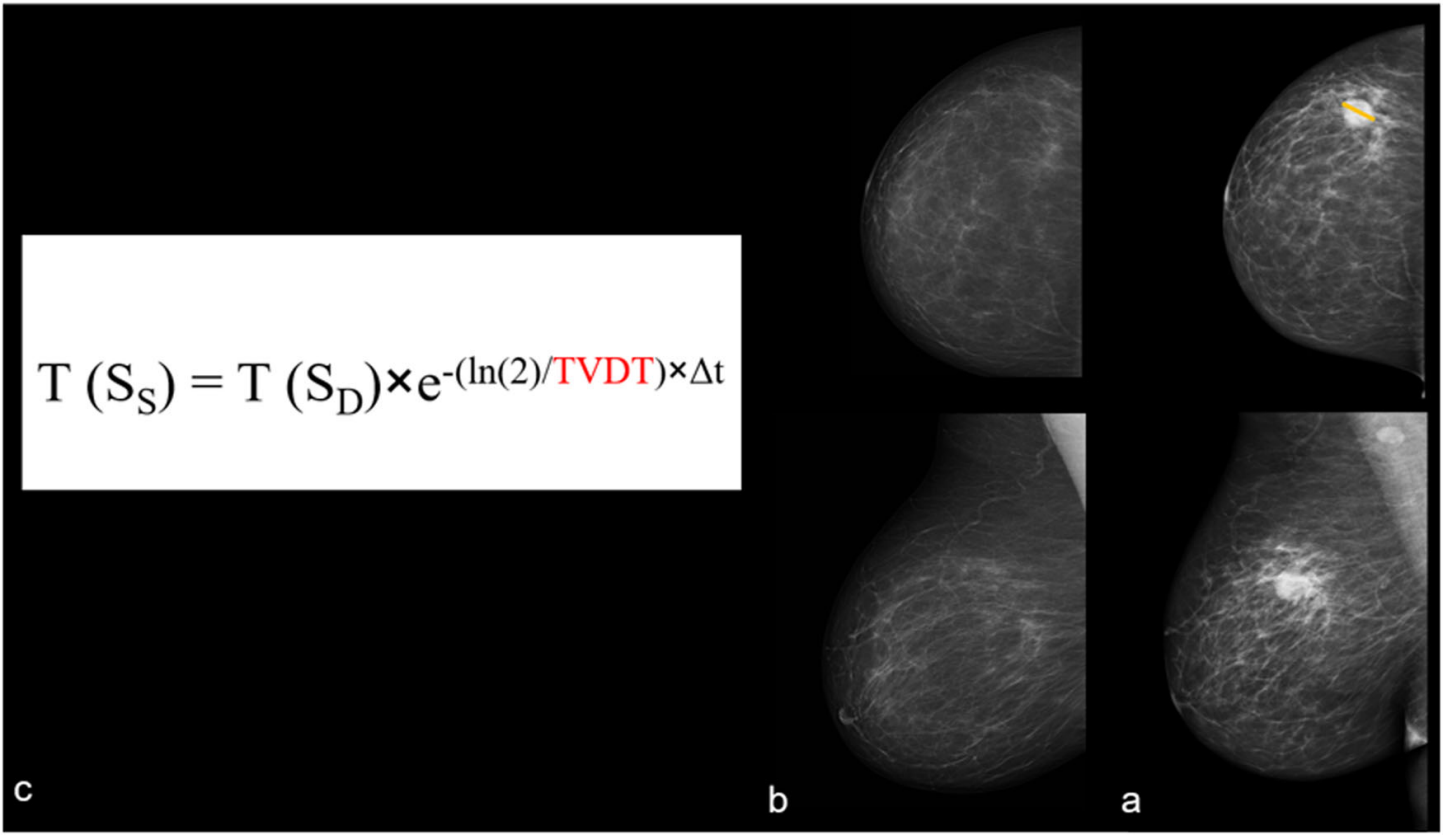
Non-visible interval cancer at the time of screening: A 66-year-old female was diagnosed in the second year following the screening (758 days) with a 25 mm grade 3 ER-negative HER2 positive IDC with HGDCIS (**a**). Based on the patient’s age, cancer grade, and ER status, an estimated TVDT of 143 days was used in the equation (red, **c**). Together with the longest tumour diameter at diagnosis (S_D_) and time interval in days (Δt), a tumour size at screening (S_S_) was estimated at 0.7 mm (**b**). HGDCI, high-grade ductal carcinoma in situ; IDC, invasive ductal carcinoma; TVDT, tumour volume doubling time

Associations between TVDT and clinicopathological variables of visible cancers (ER, HER2, grade, age at diagnosis, and breast density) were assessed using univariate analyses, and those with *p* < 0.1 were included in a generalised linear model (logarithmic link function, Gaussian distribution) to estimate TVDT for non-visible cancers (TVDT_E_). For non-visible ICs at screening, size was calculated using [T (S_S_) = T (S_D_) × e^−^^λ × Δt^], where T (S_S_) and T (S_D_) refer to the tumour size (mm) at screening and diagnosis, respectively, Δt is the time between diagnosis and screening (days), and λ = ln (2)/TVDT_E_. Figure 3 illustrates the estimation of cancer size of a non-visible cancer at screening. The time point at which non-visible cancers might have become visible was estimated using [t = −ln (S_S_/S_D_)/λ], where t is the time between diagnosis and the estimated time point for cancer visibility. S_S_ is a suggested cancer size that could be detectable at the time of screening (i.e., 5 mm if the diagnosed tumour was located in a fatty area, or 10 mm if the diagnosed tumour was located in a dense area) [14–16], S_D_ is the longest diameter of non-visible cancer at diagnosis (mm), and λ = ln (2)/TVDT_E_.

### Statistical analysis

Jamovi version 2.3 was used for data analysis. Mann–Whitney U or Kruskal–Wallis test was used to compare groups with Bonferroni correction for multiple comparisons, where appropriate. Spearman correlation coefficient (ρ) was used to evaluate the associations between continuous variables. The chi-square (χ^2^) test was used to determine associations between categorical variables. The significance threshold was set at *p* < 0.05.

## Results

### Patient characteristics

Of 666 ICs, 190 cases were excluded from the analysis, Fig. 1. After exclusions, the final dataset comprised 476 interval cancers in females, with a median age at screening of 59 years (IQR 53–65). Of the total number of ICs examined, only 79 (16.9%) ICs were diagnosed within 12 months, with 164 (34.4%) and 233 (48.9%) diagnosed within 12–24 and 24–40 months after screening, respectively. Dense breasts (BI-RADS C and D) were observed in 287/476 (60.2%) of cases. The majority of ICs were grade 2 or 3 410/476 (86.1%) and of luminal subtype 382/476 (80.2%). The histopathological cancer characteristics in different breast density groups are provided in Appendix 1.

### Interval cancer review

Out of 476 ICs, 281 were classified as visible (59%) and 195 (40.9%) as non-visible on prior screening mammograms. Younger age and longer time-to-interval were associated with non-visible cancers at screening, whereas no association was observed between tumour visibility and histopathology or breast density (Table 1).

**Table 1:** Interval cancers dataset characteristics

Characteristic	All interval cancers (*n* = 476)	Visible (*n* = 281)	Non-visible (*n* = 195)	*p*-value
Screening age (years)	59 [53–65]	60 [53–66]	57 [51.5–64.5]	0.06
Screening age group				0.03
47–59 years	262 (51.6%)	134 (47.6%)	128 (65.6%)	
60+ years	214 (48.3%)	147 (52.3%)	67 (34.3%)	
Breast density				0.27
a	28 (5.9%)	18 (6.4%)	10 (5.1%)	
b	160 (33.6%)	91 (32%)	69 (35.5%)	
c	247 (52%)	154 (54.4%)	93 (47.9%)	
d	40 (8.4%)	18 (6.4%)	22 (11.3%)	
Cancer category				NA
Normal or Benign	334 (70.1%)	139 (49.4%)	195 (100%)	
Minimal signs	118 (24.7%)	118 (41.9%)		
Suspicious	24 (5.04%)	24 (8.5%)		
Time to interval (days)	722 [473–920]	687 [425–888]	754 [511–951]	0.005
0–12 months	79 (16.5%)	54 (19.2%)	25 (12.8%)	0.032
13–24 months	164 (34.4%)	100 (35.5%)	64 (32.8%)	
25–40 months	233 (48.9%)	127 (45.1%)	106 (54.3%)	
Cancer grade				0.08
Grade1	51 (10.7%)	37 (13.1%)	14 (7.1%)	
Grade2	215 (45.1%)	124 (44.1%)	91 (46.6%)	
Grade3	195 (40.9%)	108 (38.4%)	87 (44.6)	
Missing	15 (3.1%)	12 (4.2%)	3 (1.53%)	
ER status				
Negative	82 (17.2%)	42 (14.9%)	40 (20%)	0.14
Positive	382 (80.2%)	229 (81.4%)	153 (78.9%)	
Missing	12 (2.5%)	10 (3.5%)	2 (1%)	
HER2 status				0.45
Negative	367 (77.1%)	224 (79.7%)	143 (73.3%)	
Positive	83 (17.4%)	47 (16.7%)	36 (18.4%)	
Missing	26 (5.4%)	10 (3.5%)	16 (8.2%)	
Molecular subtype				0.25
Luminal	382 (80.2%)	229 (81.5%)	154 (78.9%)	
HER2-enriched	28 (5.8%)	16 (5.7%)	11 (5.6%)	
Triple-negative	54 (11.3%)	26 (9.2%)	28 (14.3%)	
Missing	12 (2.5%)	10 (3.5%)	2 (1%)	

Continuous data are presented as medians with the interquartile range in square brackets. Categorical data are presented as absolute numbers with percentages in parentheses. Breast density was classified according to the Breast Imaging Reporting and Data System (BIRADS)

Readers classified 334/476 (70.1%) ICs as normal or benign (true negatives), 118/476 (24.7%) as minimal signs and 24/476 (5.0%) as suspicious (false negatives). The radiological features of the visible cancers at the screening included mass 58 (23.4%), distortion 68 (27.5%), asymmetry 111 (44.9%), and calcifications 10 (4.04%). A subgroup analysis of IC categories and histopathological characteristics is provided in Appendix 2.

### TVDT for visible cancers at screening

**Fig. 4:**
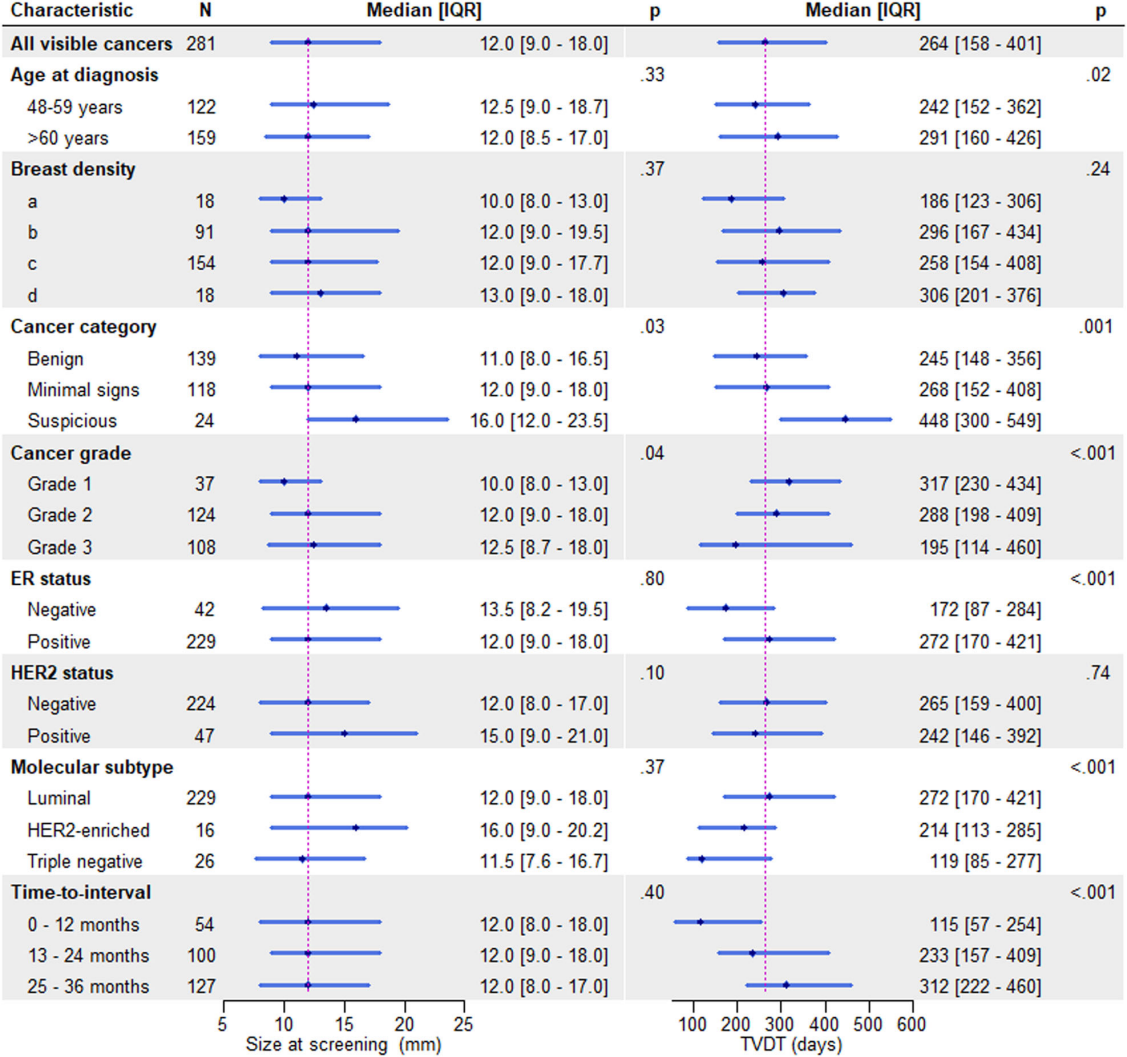
Size at screening and tumour volume doubling time (TVDT) for visible interval cancers at screening (*n* = 281) stratified by clinicopathological characteristics: The magenta lines indicate the median size at screening and TVDT for all visible cancers. The dot and the length of each horizontal line denote the median and interquartile range (IQR), respectively. Size at screening represents the longest diameter

Table 2 and Fig. 4 show the TVDT and size at screening and diagnosis, respectively, for visible cancers across clinicopathological variables. The median longest diameter at screening was 12 mm (IQR 9–18), and the median longest diameter at diagnosis was 25 mm (IQR 17–33). The median longest diameter at screening was shorter for ICs in non-dense breast areas compared to those in dense breast areas (BI-RADS A and B: 11 mm (IQR 8–15.2) vs BI-RADS C and D: 14 mm (IQR 9–18), *p* = 0.003). The median TVDT was 264 days (IQR 158–401). The median TVDT was 245 days (IQR 148–356) for cancers classified as normal or benign, 268 days (IQR 152–408) for cancers with minimal signs of malignancy, and 448 days (IQR 300–549) for cancers classified as suspicious, *p* = 0.001. TVDT was correlated with age at diagnosis (ρ = 0.14), *p* = 0.02. Shorter TVDT was observed in grade 3 than in grade 2 or 1 cancers, and ER-negative vs ER-positive tumours, *p* < 0.001. No evidence of a difference was observed between TVDT and other clinicopathological variables, Table 2. TVDT for visible cancers stratified by cancer grade and ER status is shown in Appendix 3.

**Table 2:** TVDT and size of the visible cancers at screening

Dataset characteristics	Interval cancer (visible)	Size at screening longest diameter (mm)	*p*-value	Size at diagnosis longest diameter	*p*-value	Time from negative screening to IC diagnosis (days)	*p*-value	TVDT (days)	*p*-value
Age at diagnosis^a^	62 [55–68]	12 [9–18]	0.12	25 [17–33]	0.003	687 [425–888]	0.11	264 [158–401]	0.02
47–59	122 (43.4%)	12.5 [9–18.7]	0.33	26.5 [18–36]	0.079	685 [398–874]	0.1	242 [152–362]	0.13
60+	159 (56.5%)	12 [8.5–17]		22 [16–31.5]		687 [443–895]		291 [160–426]	
Breast density			0.37		0.46		0.79		0.24
a	18 (6.4%)	10 [8–13]		22 [14–26]		689 [517–837]		186 [123–306]	
b	91 (32%)	12 [9–19.5]		24 [17–34]		723 [468–947]		296 [167–434]	
c	154 (54.4%)	12 [9–17.7]		25 [17–34]		679 [401–879]		258 [154–408]	
d	18 (6.4%)	13 [9–18]		23.5 [19–31.5]		670 [417–889]		306 [201–376]	
Cancer category			0.031		0.88		0.37		0.001
Benign	139 (49.4%)	11 [8–16.5]		25 [17–33]		691 [422–907]		245 [148–356]	
Minimal signs	118 (41.9%)	12 [9–18]		24.5 [17–36]		685 [421–852]		268 [152–408]	
Suspicious	24 (8.5%)	16 [12–23.5]		24.5 [16.7–30]		655 [455–942]		448 [300–549]	
Time to interval			0.4		0.24		< 0.001		< 0.001
0–12 months	54 (19.2%)	12 [8–18]		22.5 [14.5–32]		214 [159–307]		115 [57–254]	
13–24 months	100 (35.5%)	12 [9–18]		22 [17–32]		573 [464–656]		233 [157–409]	
25–40 months	127 (45.1%)	12 [8–17]		26.5 [17–35]		919 [821–998]		312 [222–460]	
Cancer grade			0.042		< 0.001		0.42		< 0.001
Grade1	37 (13.7%)	10 [8–13]		17 [12–20]		692 [412 –947]		317 [230–434]	0.001
Grade2	124 (46.09%)	12 [9–18]		24 [16–33]		724 [465–894]		288 [198–442]	< 0.001
Grade3	108 (40.1%)	12.5 [8.7–18]		27.5 [19–36]		661 [385–876]		195 [114–336]	
ER status									
Negative	42 (15.4%)	13.5 [8.2–19.5]	0.8	30.5 [20.5–40]	0.003	542 [280–872]	0.2	172 [87–284]	< 0.001
Positive	229 (84.5%)	12 [9–18]		23 [16–32]		691 [447–888]		272 [170–421]	
HER2 status									
Negative	224 (82.6%)	12 [8–17]	0.1	24 [16.7–32]	0.024	679 [408–876]	0.25	265 [159–400]	0.74
Positive	47 (17.3%)	15 [9–21]		28 [18.5–40.5]		764 [465–917]		242 [146–392]	
Molecular subtype									
Luminal	229 (84.5%)	12 [9–18]	0.37	23 [16–32]	0.018^b^	691 [447–888]	0.09	272 [170–421]	< 0.001
HER2-enriched	16 (5.9%)	16 [9–20.2]		35.5 [21–43.2]		793 [487–921]		214 [113–285]	
Triple-	26 (9.5%)	11.5 [7.6–16.7]		30 [21–36]		458 [250–807]		119 [85–277]	

Categorical data are presented as absolute numbers with percentages in parentheses. TVDT and time from negative screening to interval cancer diagnosis are in days. The median of the cancer size represents the median of the longest diameter in mm. The interquartile range is in square brackets [IQR]. There is a significant difference in the screening size between grade 1 and grade 2 groups, *p* = 0.04. There is a significant difference in the diagnostic size between grade 1 and the other 2 groups, but not between grade 2 and 3. The difference in tumour receptors TVDT is between luminal and triple-negative cancers, *p* = 0.001. There is a significant difference in the screening size between cancers classified as benign and cancers classified as suspicious, *p* = 0.04

^a^ Data are presented as medians

^b^ Differences between groups were found to be significant (*p* = 0.02), but pairwise group differences were not significant following correction for multiple comparisons

In a generalised model with TVDT as the dependent variable and age at diagnosis, grade, and ER status as predictors, cancer grade was the strongest variable to explain TVDT (η^2^p = 0.051), *p* = 0.001.

### TVDT estimation for non-visible cancers at screening

The equation used to calculate TVDT for non-visible ICs is given in Appendix 4. The median TVDT_E_ was 226 days (IQR 171–253). Unlike our findings in visible cancers, longer TVDT_E_ was found in women < 60 years at diagnosis vs those > 60 years (*p* = 0.003).TVDT_E_ for cancer grade and ER status is shown in Appendix 5.

The median estimated cancer size at the time of screening was 1.7 mm (IQR 0.8–3.8); 1.7 mm (IQR 1.0–2.5) for grade 1, 2.5 mm (IQR 1.5–5.9) for grade 2, and 0.9 mm (IQR 0.4–2.0) for grade 3 cancers, *p* < 0.001. The median estimated cancer size was 2.0 mm (IQR 1.0–4.7) for luminal cancers, 0.6 mm (IQR 0.3–2.1) for HER2-enriched cancers, and 0.3 mm (IQR 0.1–1.3) for triple-negative cancers, *p* < 0.001. For the cancers diagnosed in the third year following the screening, the median size at diagnosis was 21 mm (IQR 14–30) compared to 9.8 mm (IQR 6.7–15.6) estimated size at 24 months after screening.

**Fig. 5:**
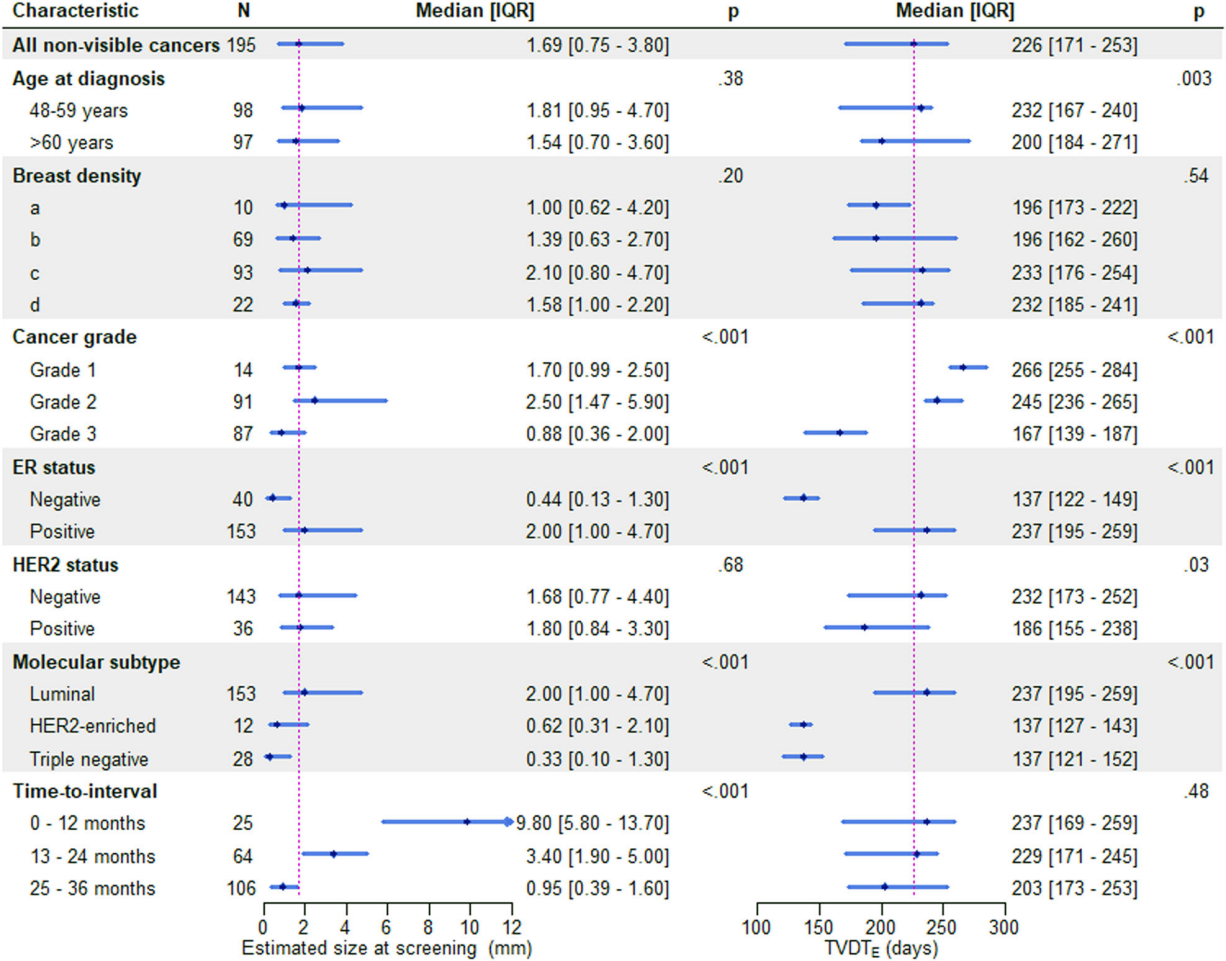
Estimated tumour volume doubling time (TVDT_E_) and size at screening for non-visible interval cancers at screening (*n* = 281) stratified by clinicopathological characteristics: The magenta lines indicate the median size at screening and TVDT for all non-visible cancers. The dot and the length of each horizontal line denote the median and interquartile range (IQR), respectively. Size at screening represents the longest diameter

At the threshold for cancer visibility (5 mm in fatty breast areas and 10 mm size in dense breast areas), the median estimated time for cancer visibility was 489 days (IQR 229–682) after the screening. This applies to 648 days (IQR 527–799) for cancers diagnosed in the third year following the screening. The analysis of TVDT_E_, cancer size at screening, and the time point of cancer visibility for the non-visible ICs are given in Table 3 and Fig. 5.

**Table 3:** TVDT, cancer size estimation at screening, and cancer detectability time for non-visible cancers at 5 mm and 10 mm cancer size cut-offs for cancers developed in fatty and dense breast areas, respectively

Dataset characteristics	Interval cancer (non-visible)	Size at screening longest diameter (mm)	*p*-value	Time from screening to cancer became visible (days)	*p*-value	Time from cancer became visible to diagnosis (days)	*p*-value	TVDT_E_ (days)	*p*-value
Age at diagnosis	59 [54–67]	1.69 [0.75–3.8]	0.047	489 [232–676]	0.06	280 [147–394]	0.66	226 [171–253]	0.02
48–59	98 (50.2%)	1.81 [0.95–4.7]	0.38	505 [166–651]	0.22	280 [178–381]	0.65	232 [167–240]	0.003
60+	97 (49.7%)	1.54 [0.7–3.6]		470 [267–736]		280 [128–416]		200 [184–271]	
Breast density			0.2		0.45		0.03^a^		0.54
a	10 (5.1%)	1 [0.62–4.2]		418 [65–549]		445 [340–501]		196 [173–222]	
b	69 (35.5%)	1.39 [0.63–2.7]		490 [262–676]		308 [197–399]		196 [162–260]	
c	93 (47.9%)	2.1 [0.8–4.7]		467 [208–655]		269 [120–380]		233 [176–254]	
d	22 (11.3%)	1.58 [1–2.2]		583 [386–714]		194 [120–270]		232 [185–241]	
Time to interval	754 [511–951]		< 0.001		< 0.001		0.29		0.48
0–12 months	25 (12.8%)	9.8 [5.8–13.7]		−4.9 [119–150]		232 [118–364]		237 [169–259]	
13–24 months	64 (32.8%)	3.4 [1.9–5]		264 [113–418]		289 [167–428]		229 [171–245]	
25–40 months	106 (54.3%)	0.95 [0.39–1.6]		645 [527–798]		268 [147–394]		203 [173–253]	
Cancer grade			< 0.001		0.018		0.002		< 0.001
Grade1	14 (7.1%)	1.7 [0.99–2.5]		603 [453–778]		101 [−3 to 203]		266 [255–284]	0.024
Grade2	91 (47.3%)	2.5 [1.47–5.9]		392 [153–616]		326 [188–446]		245 [236–265]	
Grade3	87 (45.1)	0.88 [0.36–2]		522 [287–715]		265 [142–338]		167 [139–187]	
ER status									
Negative	40 (20.7%)	0.44 [0.13–1.3]	< 0.001	591 [343–713]	0.046	206 [132–316]	0.014	137 [122–149]	< 0.001
Positive	153 (79.2%)	2 [1–4.7]		461 [219–645]		301 [157–434]		237 [195–259]	
HER2 status									
Negative	143 (79.8%)	1.68 [0.77–4.4]	0.68	502 [231–682]	0.59	284 [147–422]	0.12	232 [173–253]	0.03
Positive	36 (20.1%)	1.8 [0.84–3.3]		411 [254–590]		221 [123–321]		186 [155–238]	
Molecular subtype									
Luminal	153 (79.2%)	2 [1–4.7]	< 0.001	461 [219–645]	0.09	301 [157–434]	0.048	237 [195–259]	< 0.001
Her2-enriched	12 (6.2%)	0.62 [0.31–2.1]		499 [301–644]		189 [121–313]		137 [127–143]	
Triple-	28 (14.5%)	0.33 [0.1–1.3]		607 [363–719]		211 [137–315]		137 [121–152]	

Categorical data are presented as absolute numbers with percentages in parentheses. Continuous data are presented as medians with the interquartile range in square brackets. Estimated TVDT, the time from screening (non-visible) to cancer being visible, and the time from cancer being visible to diagnosis are in days. The estimated cancer size represents the median of the longest diameter in mm

^a^ Differences between groups were found to be significant (*p* = 0.03), but pairwise group differences were not significant following correction for multiple comparisons

## Discussion

This retrospective analysis of an 8-year cohort of consecutive interval cancers shows that almost half of invasive ICs are diagnosed in the third year after screening, that 86% are grade 2 or 3, and with hindsight, there is a visible abnormality on the screening mammogram in 59% (281/476) of cases.

The retrospective visibility of early tumour features at screening depends on the quality of images, breast density, cancer size, number of reviewers, and the review method (blinded or informed) [17, 18]. In this fully informed, consensus-based, retrospective review of screening mammograms and the corresponding diagnostic mammograms, 70.1% were classified as true negatives (139 visible and 195 non-visible), 118 (24.7%) as minimal signs, and 24 (5%) as false negatives.

For the visible cancers, the median TVDT was longer with cancers classified as false negatives (448 days) than those of minimal signs of malignancy and true negatives (268 days and 245 days, respectively), indicating that different radiological characteristics exhibited distinct growth rates.

Shorter TVDT was seen in grade 3 cancers compared to both grade 1 and 2 cancers (195, 288, and 317 days), *p* < 0.001, which is in line with previous studies [11, 18, 19].

In our population, ER-negative cancers were more rapidly growing than ER-positive tumours, which agrees with previously published studies [11, 18, 19]. Similar to MacInnes et al [11], we found no evidence of a difference in TVDT based on HER2 status, although HER2+ cancers tended to exhibit shorter TVDT compared to HER2- tumours. While this finding may be due to the limited number of HER2+ cancers in our tumour sample, it is in broad agreement with findings from previous reports [19, 20] and may be attributed to the higher proliferation rates found in HER2+ vs HER2- cancers [21].

With regard to age and TVDT, MacInnes et al found a longer TVDT for females aged above 60 at diagnosis [11], while Otten et al [14] reported no difference in TVDT with age at diagnosis in 293 consecutively diagnosed invasive ductal breast cancers from a screened 50–74-year-old population [14]. In agreement with MacInnes et al [11], we observed a positive association between TVDT and age (ρ = 0.14), *p* = 0.02. As shorter TVDT may reflect increased tumour aggressiveness [11, 19], our findings are in keeping with numerous studies linking younger age to more aggressive tumour biology [22]. In non-visible cancers, TVDTE was longer in women < 60 years compared to those > 60 years, contrasting our observations in visible cancers. This difference may be due to the composition of our non-visible cancer sample, which predominantly comprised ER+ tumours (83/98; 84.7%) in women < 60 years and grade 3 cancers (48/95; 50.5%) in women > 60 years.”

In agreement with MacInnes et al [11], we observed no statistically significant association between mammographic breast density and TVDT. While the limited number of cancers in breast areas classified as almost entirely fatty (BI-RADS A) or very dense (BI-RADS D) in our study confounded our ability to detect differences in TVDT, a trend for shorter doubling time was observed in those visible and non-visible ICs with very fatty breasts compared to the densest category. MacInnes et al [11] similarly reported a tendency for shorter TVDT in very fatty breasts, which is consistent with the previously reported association of low breast density with increased tumour aggressiveness and poorer prognosis [11, 23–25].

The use of an estimated tumour doubling time for non-visible cancers means that data from all interval cancers can be used to model changes to screening policy. The regression analysis from visible cancers in the same screening population means the estimates are likely to be more accurate than taking TVDT from those published in the literature.

From the retrospective review, the visible cancers 281/476 (59%) could potentially be detected by supplemental imaging at the time of screening. Regarding the non-visible cancers, 106/195 (54.3%) of them were diagnosed in the third year following the screening. Our results suggest that there is a potential for a great proportion of them to be detectable at a median size of 9.8 mm (IQR 6.7–15.6) at a 2-year screening interval, with a median estimated visibility time point of 645 days (IQR 527–798) after the screening.

When considering changes to screening regimes policymakers are reliant on good-quality health economic modelling to consider multiple options. The aim of screening is to find grade 2 and 3 cancers when they are still small so incorporation of TVDT and IC characteristics is important. Our study suggests that reducing frequency from three to two years is important to detect more grade 2 and 3 cancers at a small size. The large number of ICs that are “visible” on review suggests that supplemental imaging could play an important role in reducing these.

The strengths of this study are that a large number of consecutive interval cancer cases from one site were included in the analysis, and a large proportion of these showed a visible abnormality at the same location as the interval cancer. Consensus measurements by experienced breast radiologists using a previously reported accepted technique for calculating doubling time was used [12, 13]. Careful consideration was given to ensure the correct site was measured, and this was often checked with the pathology and surgical report allowing for consistency in the measurements. The estimation of TVDT in non-visible cancers was based on observations from a contemporaneous cohort of visible cancers in the same population.

The limitations of this study are that interval cancers from only one site and predominantly from one manufacturer were included. Larger multi-site studies are required to confirm our results. The tumour growth was modelled as following a constant exponential function over time, recognising that actual growth rates may vary due to other latent factors. We assumed that tumour grade does not change significantly over time, acknowledging that progression, if it occurs, is uncommon [26–28]. These assumptions are necessary for the practical application of our model and align with common practices in tumour growth estimation [11, 14, 29, 30]. Future research could further refine these estimates by incorporating additional variables and exploring more complex models.

## Conclusion

Faster tumour growth was associated with younger age and with grade 3, ER-negative, and true-negative interval cancers. A trend toward shorter TVDT was observed in fatty breasts compared to dense breasts; however, this observation was not statistically significant and may be due to chance. The TVDT estimates could be used in health economic models to better predict the benefits of supplemental imaging and the frequency of screening at a given minimum detectable size.

## Supplementary information

ELECTRONIC SUPPLEMENTARY MATERIAL

## Abbreviations

BI-RADS: Breast Imaging Reporting and Data System
CC: Craniocaudal
ER: Oestrogen receptor
HER2: Human epidermal growth factor 2
IC: Interval cancer
MLO: Mediolateral oblique
NHSBSP: National Health Service Breast Screening Programme
TVDT: Tumour volume doubling time
