# Supplementary material for: Tumour growth rate and invasive interval cancer characteristics in a UK breast cancer screening population
Source: Eur Radiol. 2025 Jan 21;35(7):4001–12. doi: 10.1007/s00330-024-11342-x (PMC12165881; doi:10.1007/s00330-024-11342-x)
Supplement: Supplementary file 1 — ELECTRONIC SUPPLEMENTARY MATERIAL [file 330_2024_11342_MOESM1_ESM.pdf]

Tumour growth rate and invasive interval cancer characteristics in a UK breast cancer screening population

ELECTRONIC SUPPLEMENTARY MATERIAL

Appendix 1

Subgroup analysis of histopathological interval cancer characteristics and breast density

The proportion of grade 3 cancers was 91/179 (50.8%) in non-dense breasts and 103/281 (36.6%) in dense breasts, $P = 0.009$. This applies to 14/26 (53.8%) in very fatty breasts, and 10/40 (25%) in the densest breasts, $P = 0.015$. The proportion of triple-negative cancers was 29/181 (16%) in non-dense breasts and 24/282 (8.5%) in dense breasts, $P = 0.026$. This applies to 3/26 (11.5%) in very fatty breasts and 3/40 (7.5%) in the densest breasts, $P = 0.24$.

Appendix 2

Subgroup analysis of histopathological characteristics and interval cancer categories
Of 82 ER-negative cancers, 65/82 (79.2%) were classified as normal/ benign, and 17/82 (20.7%) were classified as minimal signs, while 23/23 cancers classified as false negatives were ER-positive, *P* = 0.036. Grade 3 cancer comprised 147/325 (45.2%) of true negatives, 46/114 (40.3%) of cancers with minimal signs, and 2/22 (9.1%) of false negatives. Of the grade 3 cancer group, 147/195 (75.3%) were true negative, 46/195 (23.5%) had minimal signs, and 2/195 (1%) were false negative, *P* = 0.006.

Appendix 3

	ER-negative	ER-positive	Either ER	p-value
**Number**				
Grade1	0	37	37	
Grade2	9	114	123	
Grade3	32	76	108	
Any grade	41	227	268	
**TVDT (Median)**				
Grade1	NA	317 [230–434]	317 [230–434]	
Grade2	260 [122–296]	291 [199–457]	285 [197–443]	
Grade3	137 [73–255]	218 [132–356]	195 [114–336]	<0.001
Any grade	170 [87–281]	272 [170–419]	258 [156–396]	
**p-value**	<0.001			

Table S1: Subgroup analysis of TVDT for cancer grade and ER status for visible cancers at screening. The interquartile range is in square brackets [IQR]. There is a statistically significant difference between ER-positive and ER-negative groups, $P < 0.001$. There is a statistically significant difference between grade 3 and 2, $P < 0.001$, and between grade 3 and 1, $P = 0.0002$, but not between grade 1 and grade 2 cancers, $P = 1$. There is a statistically significant difference between ER-positive grade 3 and ER-positive grade 2, $P = 0.007$. There is a statistically significant difference between ER-negative grade 3 and ER-positive grade 2, $P < 0.001$.

Appendix 4

TVDT estimation for non-visible interval cancers

TVDT for non-visible interval cancers ($TVDT_E$) was estimated using a generalised linear model (logarithmic link function, Gaussian distribution):

$\text{Log}\ (TVDT_E) = \beta_0 + \beta_1 \times \text{Age} + \beta_2\ (\text{ER}) + \beta_3\ (\text{Grade})$

Where Age refers to the patient age at diagnosis (years), ER is the ER status of the tumour (i.e., negative or positive), Grade is the cancer grade (i.e., grade 1, 2 or 3), β_0 is the model intercept, and $\beta_1, \beta_2, \beta_3$ are the model coefficients for Age, ER status and Grade, respectively. Log refers to the natural logarithm.

For ER-negative tumours,

$\text{Log}\ (TVDT_E) = 4.73 - 0.011 \times \text{Age} - 0.184 + \beta_3\ (\text{Grade})$

For ER-positive tumours,

$\text{Log}\ (TVDT_E) = 4.73 - 0.011 \times \text{Age} + 0.175 + \beta_3\ (\text{Grade})$

With $\beta_3 = 0$ for grade 1 (reference); $\beta_3 = -0.0375$ for grade 2; $\beta_3 = -0.397$ for grade 3.

Appendix 5

	ER-negative	ER-positive	Either ER	p-value
**Number**				
Grade1	0	14	14	
Grade2	7	84	91	
Grade3	32	54	86	
Any grade	39	152	191	
**TVDT (Median)**				
Grade1	NA	266 [255–284]	266 [255–284]	
Grade2	187 [177–189]	248 [237–265]	245 [236–265]	
Grade3	131 [121–140]	181 [170–196]	167 [139–187]	<0.001
Any grade	137 [122–149]	237 [195–259]	226 [171–253]	
**p-value**	<0.001			

Table S2: Subgroup analysis of $TVDT_E$ for cancer grade and ER status for the non-visible cancers at screening. The interquartile range is in square brackets [IQR].

Appendix 6

Dataset Characteristics	Visible	Non-visible	p-value
**TVDT**	264 [158–401]	226 [171–253]	<0.001
48–59	242 [152–361]	232 [167–240]	
60+	291 [160–430]	200 [184–271]	
**Breast area density**			<0.001
Non dense	144 (51.2%)	61 (31.2%)	
Dense	123 (43.7%)	134 (68.7%)	
**Cancer size at screening**	12 [9–18]	1.69 [.75–3.8]	<0.001
**Cancer size at diagnosis**	25 [17–33]	20.8 [14.2–30]	.01

Table S3: Comparison between visible and non-visible cancers: TVDT is in days. The cancer size represents the median of the longest diameter in mm. Percentages are presented in brackets (%) and the interquartile range is in square brackets [IQR]. For non-visible cancers at screening, TVDT was estimated, time from cancer visibility to interval was estimated based on the density of the breast area, and cancer size at screening was also estimated. There is a significant difference in the estimated $TVDT_E$ between the age groups, $P = 0.005$.
